# Landmark-based registration of a cochlear model to a human cochlea using conventional CT scans

**DOI:** 10.1038/s41598-023-50632-0

**Published:** 2024-01-11

**Authors:** Jenny Stritzel, Amir Hossein Ebrahimzadeh, Andreas Büchner, Heinrich Lanfermann, Michael Marschollek, Dominik Wolff

**Affiliations:** 1 Peter L. Reichertz Institute for Medical Informatics of TU Braunschweig and Hannover Medical School, Hannover, Germany; 2 German Hearing Center, Hannover Medical School, Hannover, Germany; 3 Department of Otorhinolaryngology, Hannover Medical School, Hannover, Germany; 4 Institute of Diagnostic and Interventional Neuroradiology, Hannover Medical School, Hannover, Germany

**Keywords:** Medical research, Outcomes research, Computed tomography, Computational science

## Abstract

Cochlear implants can provide an advanced treatment option to restore hearing. In standard pre-implant procedures, many factors are already considered, but it seems that not all underlying factors have been identified yet. One reason is the low quality of the conventional computed tomography images taken before implantation, making it difficult to assess these parameters. A novel method is presented that uses the Pietsch Model, a well-established model of the human cochlea, as well as landmark-based registration to address these challenges. Different landmark numbers and placements are investigated by visually comparing the mean error per landmark and the registrations’ results. The landmarks on the first cochlear turn and the apex are difficult to discern on a low-resolution CT scan. It was possible to achieve a mean error markedly smaller than the image resolution while achieving a good visual fit on a cochlear segment and directly in the conventional computed tomography image. The employed cochlear model adjusts image resolution problems, while the effort of setting landmarks is markedly less than the segmentation of the whole cochlea. As a next step, the specific parameters of the patient could be extracted from the adapted model, which enables a more personalized implantation with a presumably better outcome.

## Introduction

The “invisible disability”, as hearing loss is often called, caused the third largest number of years lived with disability globally in 2021. The World Health Organization estimates that over 1.5 billion people of all ages, ethnicities, and economies are living with some degree of hearing loss, which affects their mental and cognitive health and daily quality of life^1^. These numbers are increasing every year^2^. The treatment of hearing impairment highly depends on the degree of hearing loss. For many affected persons, conventional hearing aids are a satisfactory solution. Still, for many affected persons, these devices cannot provide sufficient acoustic amplification to generate a benefit for everyday life^2^. This includes people who are $100\%$ deaf and those with residual hearing but whose hearing with hearing aids is no longer adequate for everyday life. Cochlear implants are mainly indicated for candidates with sensorineural hearing loss to bypass damaged hair cells. It is important that the auditory nerve is still intact. Here, another approach needs to be taken. A more advanced treatment option to restore hearing are cochlear implants (CIs). CIs overcome the limitation of traditional amplification by directly stimulating the auditory nerve fibers inside the cochlea^3^.

The appearance of the human cochlea is well described in the literature. However, there is still a lack of knowledge about which characteristics lead to optimal or poor postoperative hearing results. The success of postoperative hearing depends on various factors like anatomical properties of the cochlea itself, for example, cochlear duct length, volume, basal diameter, or malformations. The appropriate electrode length is selected, for example, to prevent any surgical injuries caused by electrodes that are too long and to improve the hearing result^4^. However, only a few of these preoperative characteristics, such as cochlear duct length, have been adequately studied, whereas the role of others, such as volume, has not yet been conclusively determined^5,6^. Generally, more extensive studies would be required for a well-founded statement in this regard. Besides natural factors, postoperative features such as CI location and penetration depth in the inner ear are also assumed to play a role^4,7,8^. On the other hand, it would greatly benefit patient care to have a prediction for postoperative hearing in a preoperative stage. As a standard procedure, computer tomography (CT) scans are taken before each implantation, which can provide information about the characteristics of the CI candidate’s cochlea that would be essential for such a prediction. A challenge arises from the low resolution of conventional CT images compared to the structural size of a human cochlea. Therefore, literature typically focuses on $\mu$CT (ex vivo) or ultra-high-resolution CT images^5,9,10^, which are mainly used for scientific studies and have somewhat less relevance in the clinical routine of cochlear implantation. There is a publicly available $\mu$CT dataset published by Gerber et al.^11^. Some papers implement a segmentation procedure of the cochleae based on $\mu$CTs and machine learning algorithms and then test it on conventional CTs in an additional step^12–15^. This requires large amounts of data in high-resolution quality. However, this work aimed to develop a solution for conventional CT data from clinical routine. In addition, a method should be developed that does not require large amounts of data. Larger amounts of data can nevertheless be examined with this method.

The comparably inferior image quality requires extensive preprocessing before the crucial features for predicting the postoperative hearing outcome can be extracted. Previous work has focused on processing retrospectively available scans of CI candidates^16^. Nevertheless, an accurate segmentation of the cochlea that is realistic in terms of volume in conventional CT scans is effectively impossible to perform due to the proportion of their average resolution (0.3 mm in all three dimensions) compared to the cochlear size (9 mm $\times \,7$ mm $\times \,4$ mm)^10^. To increase the accuracy of the segmentation, a well-evaluated model of the human cochlea developed by Pietsch et al.^9^ was adapted to the cochlea of the CI candidate using landmark-based registration. There are other models in the literature, such as Escude et al.^17^ and Schurzig et al.^18,19^. The Pietsch model offers the possibility of standardized and automated extraction of preoperative anatomical features, such as duct length, volume, or basal diameter. The code for this model is freely available. In the following, the results of an iterative process are presented to find the optimal parameters for registering the cochlear model to the segmented cochlea. In addition to the number of landmarks, the input parameters for the cochlear model are also adjusted so that better registration and smaller errors can be achieved compared to using a mean cochlea. Furthermore, to minimize the preprocessing steps, an approach is presented to perform landmark-based registration using direct landmarks from CT without prior segmentation of the human cochlea.

## Results

**Table 1:** Overview of the different examples of the number and location of landmarks (LM) on the segmented cochlea (Case 1–13) and directly in the CT data (Case 14–15). It should be noted that the choice of the best registration depends not only on the remaining error but also on the visual assessment of the result. The mean absolute error (MAE) is specified in mm per landmark.

	Description	Number of Landmarks	MAE (in mm)
Case 1 (segment)	Mean cochlea without adjustments, landmarks all $90^\circ$	12	0.2383
Case 2 (segment)	Adjustment of $A_a,\,A_b,\,B_a$ and $B_b$	12	0.2307
Case 3 (segment)	Additional adjustment of number of turns (2.4)	11	0.2106
Case 4 (segment)	Adjustments like Case 3, landmarks all $180^\circ$	6	0.1339
Case 5 (segment)	Adjustments like Case 3, landmarks all $45^\circ$	20	0.2201
Case 6 (segment)	Like Case 5, deleted 3 landmarks	17	0.1685
Case 7 (segment)	Like Case 6, deleted 2 more landmarks	15	0.1444
Case 8 (segment)	Like Case 7, deleted 2 more landmarks	13	0.1204
Case 9 (segment)	Like Case 8, deleted 1 more landmark	12	0.1075
Case 10 (segment)	Like Case 9, deleted 1 more landmark	11	0.0918
Case 11 (segment)	Like Case 10, deleted 1 more landmark	10	0.0783
Case 12*a* (segment)	Like Case 11, deleted 1 more landmark (LM 10)	9	0.0630
Case 12*b* (segment)	Like Case 11, deleted 1 more landmark (LM 2)	9	0.0663
Case 13 (segment)	Like Case 5, only first 4 and last landmark	5	0.0414
Case 14 (CT)	Like Case 3	11	0.1957
Case 15 (CT)	Like Case 13, only first 4 and last landmark	5	0.0608

**Figure 1:**
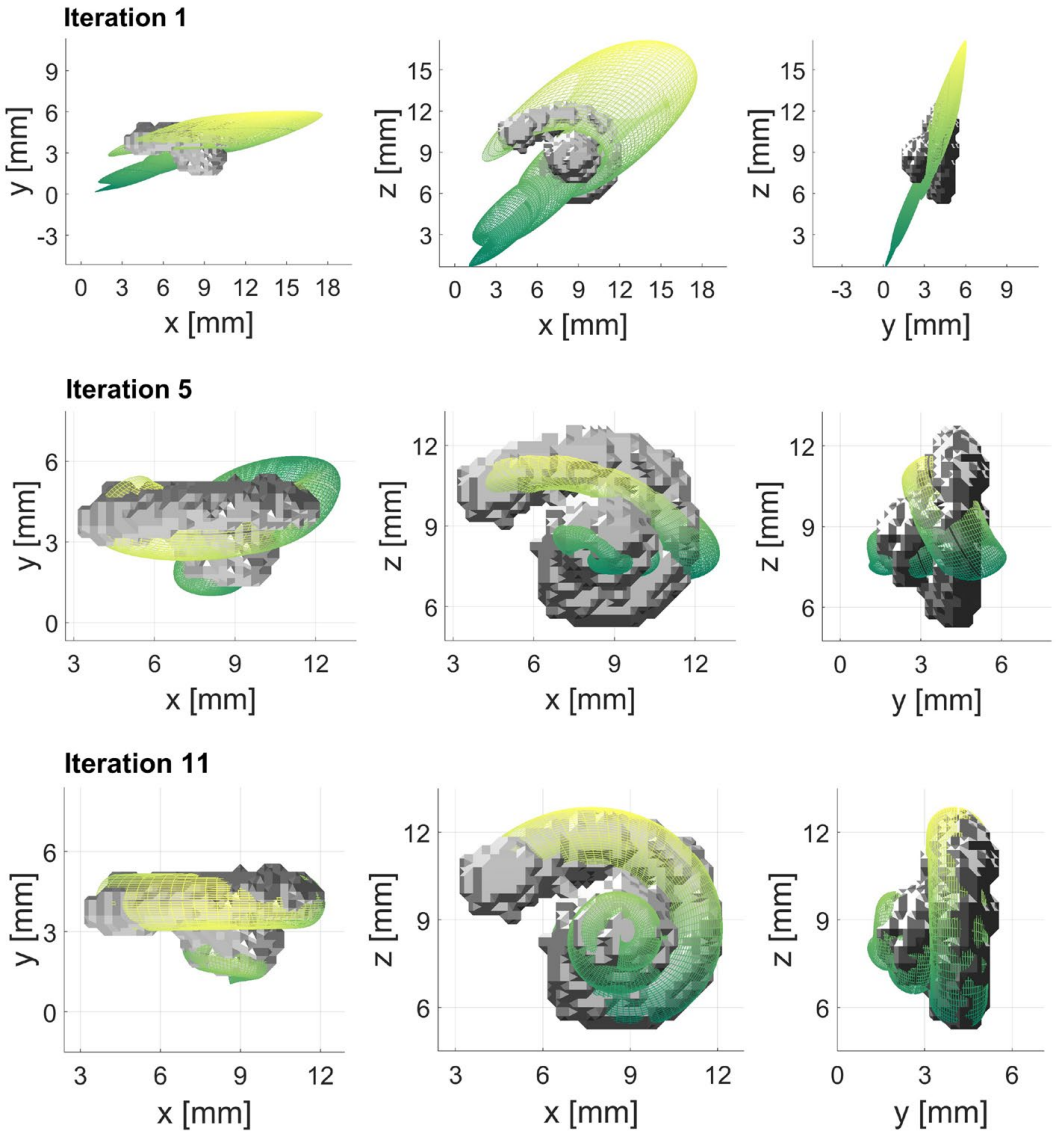
Iteration steps of landmark-based registration of cochlear model and segmented human cochlea using 12 landmarks each (Case 1). Here, the iterations 1, 5, and 11 are shown in the different planes, where the 11th iteration is the final result in this example.

The study was performed on data recorded at the *German Hearing Center Hannover*, where more than 10,000 patients (adults and children) have been fitted with a cochlear implant since 2003^20^. In this work, all experiments were performed on retrospective, preoperative CT data from one adult to show the general feasibility of the method and to find the best constellation of number and position of landmarks. In an initial experiment, the feasibility of registering the cochlear model with a segmented cochlea was evaluated^21^. In a first attempt, the model is chosen to be an average cochlea (as described in Pietsch et al.^9^) in both size and shape. Twelve corresponding landmarks were placed almost equidistantly (every $90^\circ$) on both the model and the segmented cochlea (see methods, Fig. 9). During the registration process, the model converges progressively to the size and shape of the segmented cochlea with each iteration step. In Fig. 1 shows the selected iteration steps and the corresponding results. After iteration 1, the model already moved very clearly in the direction of the segmented cochlea, and after iteration 5, a marked trend of approximation can already be seen. Iteration 11 is the final result of the landmark-based registration, which has reached its minimum mean absolute error (MAE) of 0.2383 mm for this example. This is the case when the value for the sum of squared differences (SSD) no longer changes meaningfully, and the change falls below the tolerance value $10^{-6}$. Different numbers of landmarks cause different results. To determine the optimal number of landmarks, various cases were examined. The idea behind this was that perhaps an even better arrangement and number of orientation points might be identified. To further improve the results, different examples of landmark-based registration are performed (see Table 1). In each case, the description, the number of landmarks placed, and the MAE are indicated for comparison. The number of iterations has not been specified in the table since these differ only marginally with values of 11 to 19 iterations.

**Figure 2:**
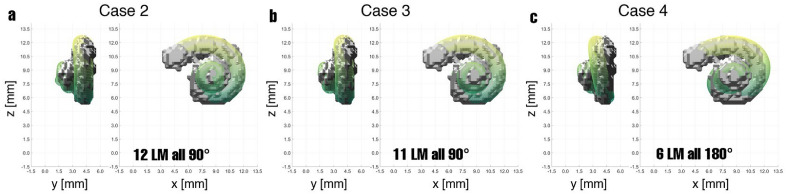
Here, the results of Cases 2, 3, and 4 are shown where the parameters of the cochlear model were adjusted. In Case 3 the number of turns was additionally adjusted. In Case 4 the landmarks are set every $180^\circ$ instead of every $90^\circ$.

**Figure 3:**
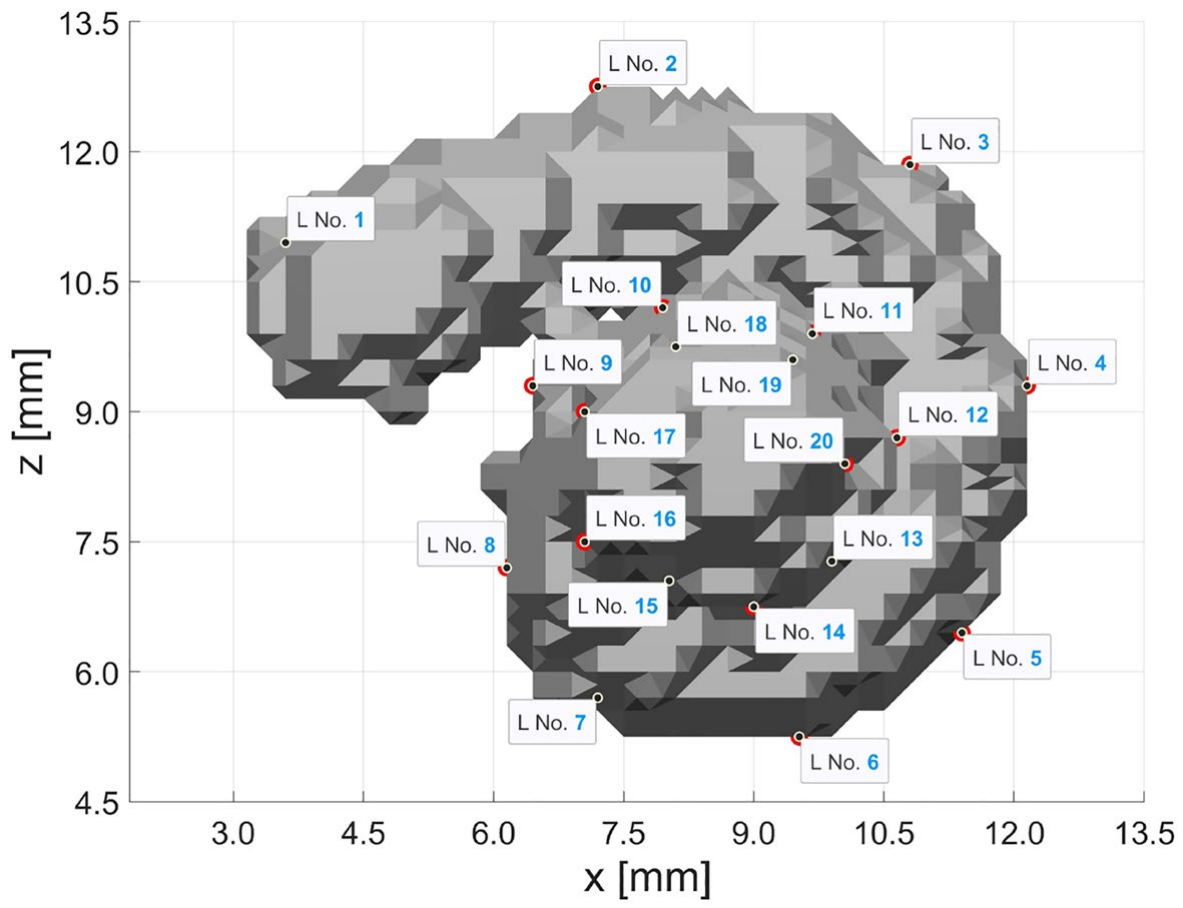
Landmark arrangement of Case 5, 20 landmarks were set every $45^\circ$.

**Figure 4:**
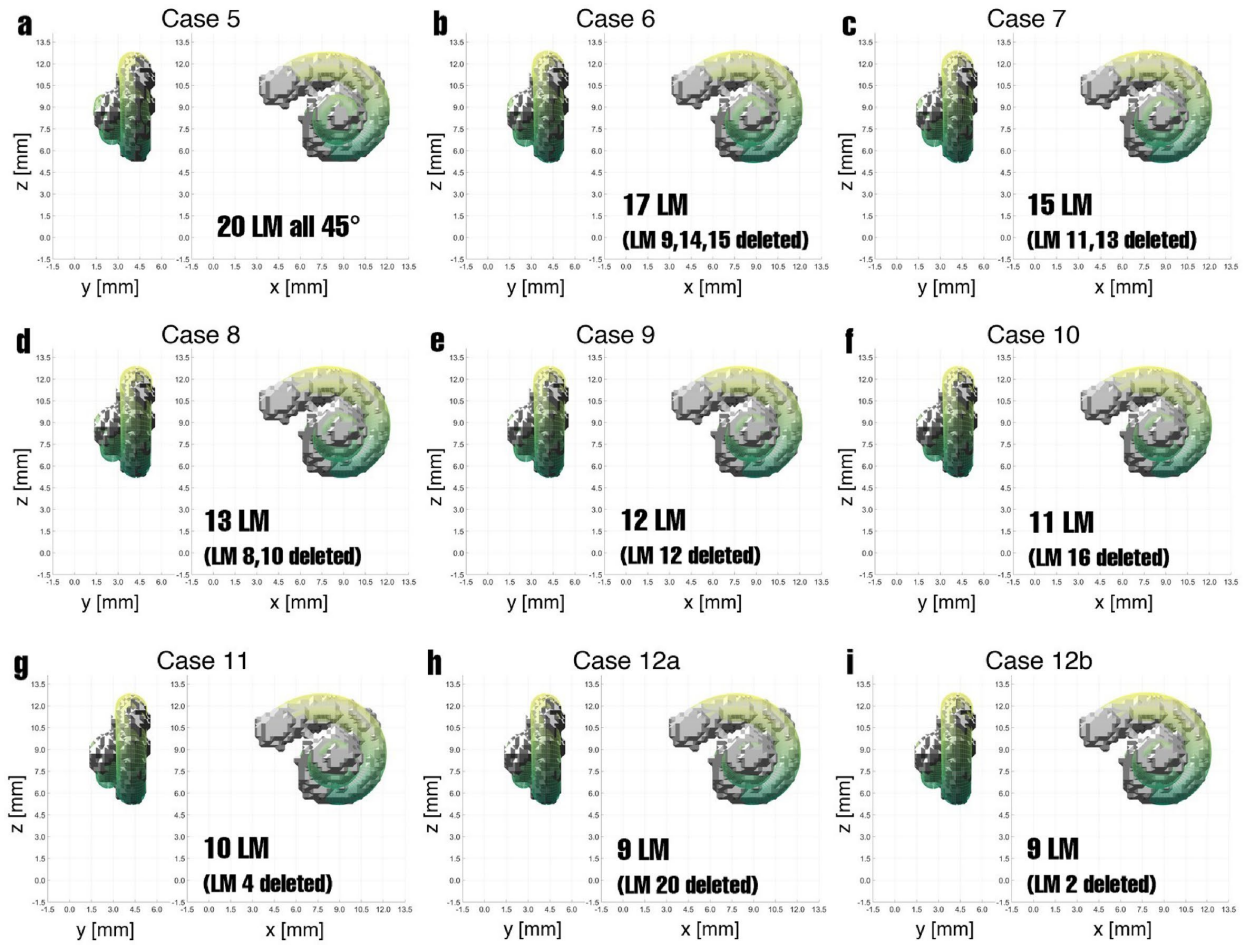
Results of the landmark-based registration for the Cases 5 to 12*b*, where different numbers of landmarks were set. Starting from Case 5, in each further case additional landmarks are deleted, which are named in the images. In most cases, only slight differences can be seen between the cochlear model and segmented cochlea.

Case 1 is the very first run with 12 set landmarks every $90^\circ$. Based on this first example, a substantial error reduction should be achieved. The first step was to adjust the parameters of the cochlear model in Case 2. For this purpose, the parameters of the four quadrants were determined as shown in Fig. 11 resulting in: $A_a = 5.71$ mm, $A_b = 3.29$ mm, $B_a = 4.37$ mm, and $B_b = 2.75$ mm for the human cochlea in this example. The landmarks are modified to fit the adjusted shape of the customized cochlear model, but the general positions every $90^\circ$ are retained.

In Case 3, the number of turns was additionally adjusted according to the anatomy of the segmented cochlea used. The number of turns was estimated to be 2.4. The landmarks on the segmented cochlea and the cochlear model remained identical, with two exceptions. Landmark No. 12 is dismissed due to the lower number of turns, and landmark No. 11 is now located at the apex. With this adjustment, the error can be further reduced. The results of Cases 2 and 3 are shown in Fig. 2a and b.

Beyond this, however, other adjustments must be investigated, especially the landmark setting. As a basis, the adjustments from Case 3 are used. In Case 4, only six corresponding landmark pairs were set every $180^\circ$. The MAE for this case is markedly reduced to 0.1339 mm per landmark compared to the previous cases. However, visual inspection of the registration’s results (Fig. 2c) shows a remarkable deviation compared to the earlier cases, especially in the top right quadrant of the *x*–*z*-plane.

**Figure 5:**
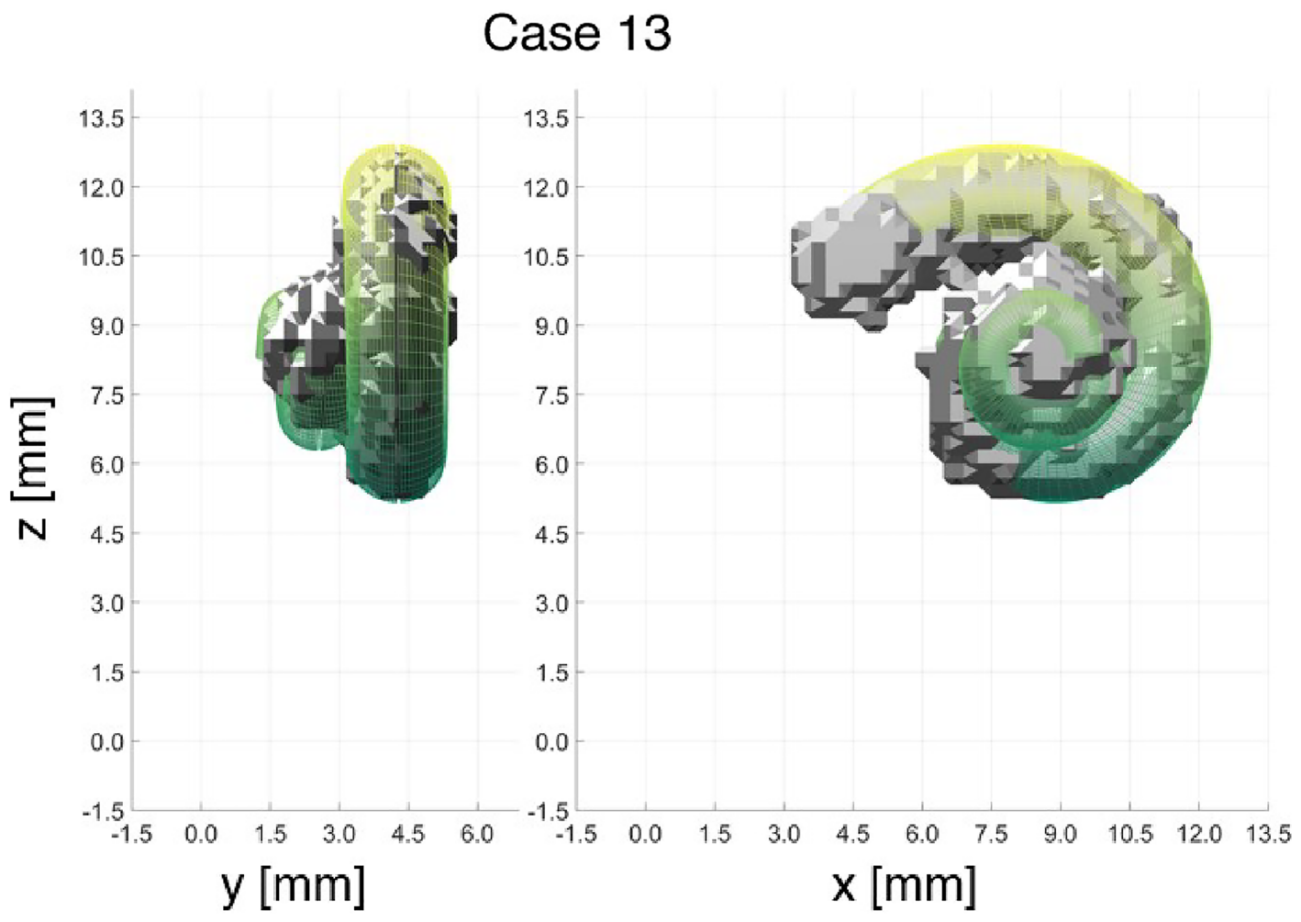
In Case 13 only 5 landmarks were set, the first four landmarks on the first level and the last landmark on top at the end of the last turn. Visually, this realization has only a little difference from the previous cases.

**Figure 6:**
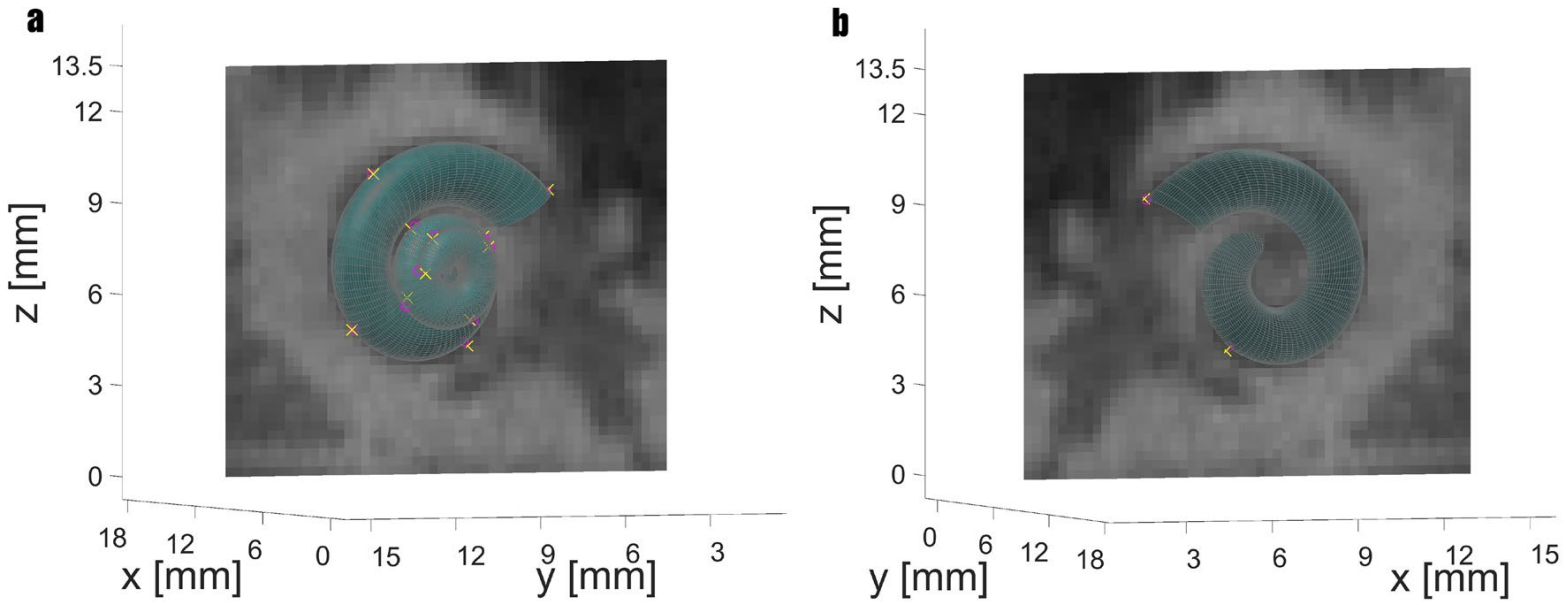
Cochlear model fitted directly to 3D CT data of the human cochlea in Case 14 (only one CT slice shown here). Model landmarks are presented in magenta circles and CT landmarks are in yellow crosses.

The results of the other Cases 5 to 12*b* are shown in Fig. 4 in the *x*–*z*-plane and *y*–*z*-plane. Here, it starts with the other extreme of many more landmarks in Case 5. It is evaluated with 20 corresponding landmark pairs. Starting with the adjustments and original landmarks from Case 3, additional landmarks were added between every two landmarks so that in the end, there is a landmark approximately every $45^{\circ }$. The exact location of the 20 landmarks can be seen in Fig. 3. Here again, the MAE has decreased compared to Case 3. In each of the following cases, landmarks producing the most substantial error are iteratively deleted. Figure 4 names the deleted landmarks that relate to the landmark numbers from Fig. 3. Furthermore, only the additionally deleted landmarks to the previous case are named in each case. Cases 12*a* and 12*b* both refer to Case 11. Thus, in Case 6, the three landmarks that caused the most substantial errors in Case 5 were deleted (leaving 17 landmarks). In Cases 7 and 8, two landmarks are deleted. Here again, as expected, an error reduction is observed. In each step, the deleted landmark position and the final visual result of the registration were analyzed to detect and prevent a meaningful deviation similar to Case 4. As long as no abnormalities occurred, this procedure was continued.

For Cases 9 to 12*b*, the landmark reduction is small-stepped, and only one landmark, i.e., the one causing the most prominent error, is deleted to avoid missing the point in time when the visual result has deteriorated too much. Both Case 12*a* and 12*b* are based on Case 11 and one landmark was deleted in each case. However, the deleted landmarks differ in position for these cases. While Case 12*a* deletes the last landmark on the apex, which produces the largest error, Case 12*b* deletes the landmark with the second largest error located on the first turn. All values for the MAE can be seen in Table 1 and all visual results in Fig. 4.

The least number of landmarks for landmark-based segmentation was investigated. Therefore, Case 13 was created, considering everything previously learned. Therefore, only 5 landmarks were defined. The first four landmarks are at the first turn and define the four input parameters of the cochlear model. The fifth landmark is at the apex of the last turn and defines the height. The result of this case is shown in Fig. 5 and shows only slight deviations from the results of the previous cases.

Additionally, it would be desirable to generally dispense with the time-consuming preprocessing with image enhancement and segmentation^16^. Therefore, the prior segmentation of the cochlea should be eliminated so that the landmarks are placed directly into the rotated CT data. In Cases 14 and 15, this is precisely what was done. Only two landmark constellations from the previous experiments are applied here. On the one hand, the basic constellation with adjusted parameters and turns, in which the landmarks are set every $90^\circ$, and on the other hand, the constellation that previously proved to be the best. Case 14 reproduces the same example as Case 3, except that this time, the landmarks were placed directly into the CT on the lateral wall of the cochlea. The MAE achieved here is 0.1957 mm per landmark and thus slightly lower than in Case 3. Figure 6 shows the transformed cochlear model within a CT slice at the level of the first four landmarks. For reasons of clearness, only a representative CT slice is shown here and not a 3D view, as the surrounding tissue is very distracting.

**Figure 7:**
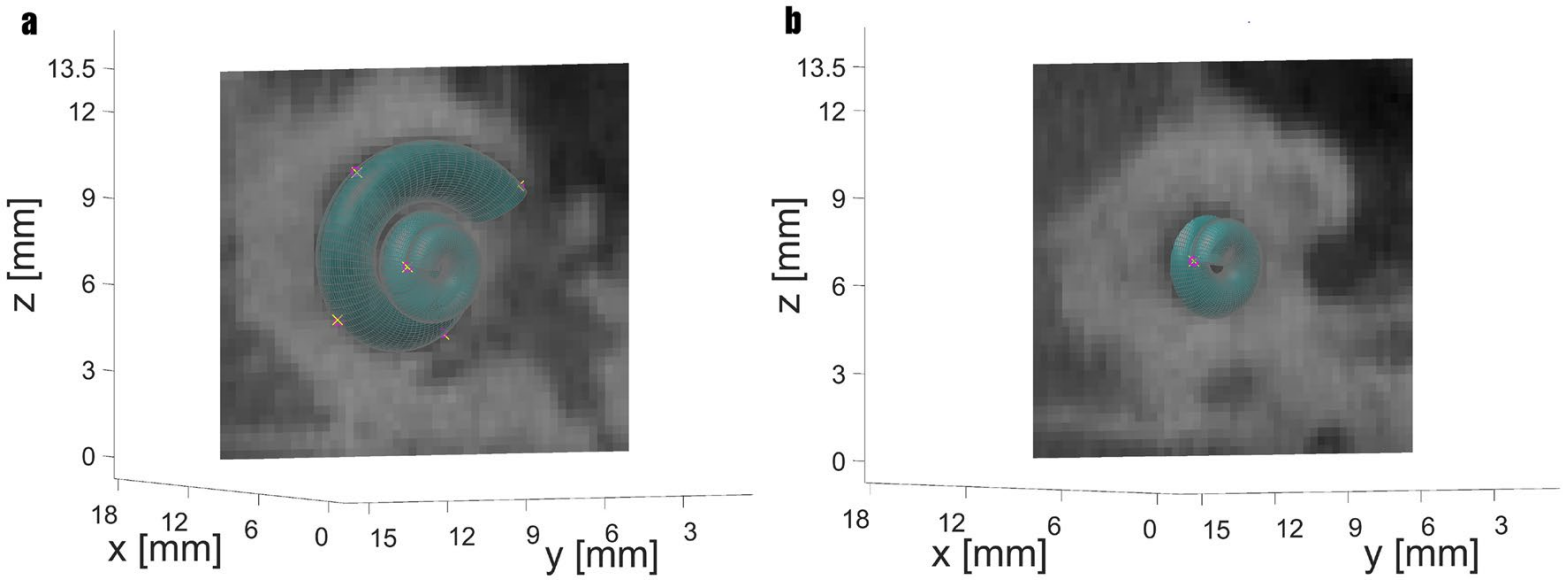
Case 15 with five set landmarks. **a** CT slice at the level of the first four landmarks. **b** Another CT slice in the middle of the cochlea.

In a second CT example (Case 15), Case 13 is replicated, and 5 landmarks are placed on the CT, four at the lowest level and the last one at the top. The MAE here is only 0.0608 mm per landmark; the result can be seen in Fig. 7. In addition to the CT slice at the level of the first four landmarks, another CT slice in the middle of the cochlea is shown in Fig. 7b. Landmarks are labeled the same as before.

## Discussion

In this work, the general feasibility of landmark-based registration of a cochlear model to an individual human cochlea segmented from CT data was tested and qualitatively evaluated. The goodness of fit as a function of the number and position of landmarks was investigated. The question is whether there is an optimal number and arrangement of landmarks. Furthermore, a way was sought to avoid the complex preprocessing of CT data and segmentation of the human cochlea, which is both very time-consuming and a potential source of error due to the low image resolution. On this occasion, the shape and volume of the human cochlea can easily be changed unintentionally by thresholding and segmentation.

In this study, Case 1 is the base case with 12 landmarks set every $90^\circ$ along the lateral wall, with the 12th landmark set at the apex ($< 90^\circ$). The model depicts an average cochlear shape. Here, an MAE of 0.2383 mm per landmark was achieved. This means that the model landmarks are, on average, 0.2383 mm distant from the human cochlear landmarks in 3D space. With a resolution of the CT data of 0.3 mm in all directions, this is already an error below the resolution of conventional CT scans. For comparison, an ultra-high-resolution CT has a resolution of approximately 0.045 mm $\times \,0.045$ mm $\times \,0.25$ mm, and a cochlea is approximately 9 mm $\times \,7$ mm $\times \,4$ mm in size^10^. Although ultra-high resolution CT scanners are most commonly used in the literature to study cochlear properties, conventional CT scanners are almost exclusively used in routine clinical practice. Most clinics perform preoperative and postoperative CT scans during cochlear implant surgery. For this reason, a method should be developed that allows the characteristics of the human cochlea to be identified from these very conventional CT scans. First and foremost, this should be made usable so that it can be integrated into the clinical workflow.

To get to this point, a few things need to be considered beforehand. The qualitative evaluation of the landmark-based registration cannot be based solely on the error deviation of the landmarks, because the visual assessment of how well the model fits the human cochlea is also essential. In Fig. 1, in addition to iteration steps 1 and 5 of Case 1, the final result after iteration step 11 can be seen. With each iteration step, the model approaches the segmented cochlea until both are reasonably well superimposed. Also, from the visual point of view, the result is impressive. Only the number of turns seems larger in the model than in the segmented cochlea. On this basis, improvements were sought both in the MAE and visually by adjusting the number of landmarks and the basic settings of the cochlear model.

Case 2 was therefore used to investigate the effect of providing the cochlear model with known values for $A_a$, $A_b$, $B_a$, and $B_b$ from the segmented cochlea. It was expected that the proportions of the four quadrants would be more consistent with the segmented cochlea. Since only affine transformations are used here, these cannot accomplish this balancing. For example, elastic transformations could be used for this purpose, but this was explicitly omitted in these experiments. Too large and for a cochlea rather untypical deformations should be avoided, so that here only the affine transformations were used. The number of 12 landmarks was retained for the time being, and these were again placed at every $90^\circ$ on the lateral wall. This case is seen in Fig. 2a and has a slightly reduced MAE of 0.2307 mm per landmark, corresponding to an error reduction of $3.19\%$. Visually, the model also appears to fit the segmented cochlea slightly better than in Case 1. Still, the difference between Cases 1 and 2 is not meaningful, as also evidenced by the difference in MAE.

Additionally, in the next step, the number of turns is adjusted in Case 3 since it seems too large in the previous cases, as indicated by the fact that the model’s apex protrudes beyond the segmented cochlea’s apex. The number of turns is reduced to a value of 2.4, which was estimated graphically based on the segmented cochlea. Due to the lower number of turns, the last landmark is omitted so that the 11th landmark is now the last one and is placed at the end of the last turn. All other landmarks remain in the same positions as before. This intervention reduces the MAE by an additional $8.71\%$ to 0.2106 mm per landmark. The resulting fit seems to have improved, with the model no longer protruding above the segmented cochlea (Fig. 2b).

These two modifications alone reduced the original MAE by $11.62\,\%$. Also, there is an improvement in the sense that the apex of the model no longer looks beyond the apex of the segmented cochlea. However, the question arises whether the number and position of the landmarks can be improved. For this reason, two other procedures of landmark setting were investigated in the next step. In Case 4, the landmarks were only set every $180^\circ$, while in Case 5, they were set every $45^\circ$ along the lateral wall. The adjustment of the parameters and the number of turns from Case 3 were kept.

In Fig. 2c shows the result of the registration in Case 4 with 6 landmarks. The MAE has decreased and is now only 0.1339 mm per landmark, but visually, the result has changed markedly. In the *x*–*z*- and the *y*–*z*-plane, the cochlear model deviates clearly from the segmented cochlea. Thus, the result is insufficient, even though the error is minimal. In both planes, especially in the region at $z = 9$ mm, the model no longer corresponds to the segmented cochlea but protrudes here.

Case 5, with 20 landmarks, is more promising and provides a much better visual result (see Fig. 4a), even though the MAE of 0.2201 mm per landmark is much larger compared to Case 4. However, the error is not markedly larger than in Case 3 despite the higher number of landmarks. Overall, the possibility of inaccuracies increases with the number of landmarks, which results from the minimization function (equation (2)^23^): setting landmarks is not a trivial task due to the poor resolution and size of 0.3 mm $\times$ 0.3 mm $\times$ 0.3 mm per voxel. It makes a big difference if a landmark is off by even one voxel in any direction, which can lead to substantial inaccuracies.

**Figure 8:**
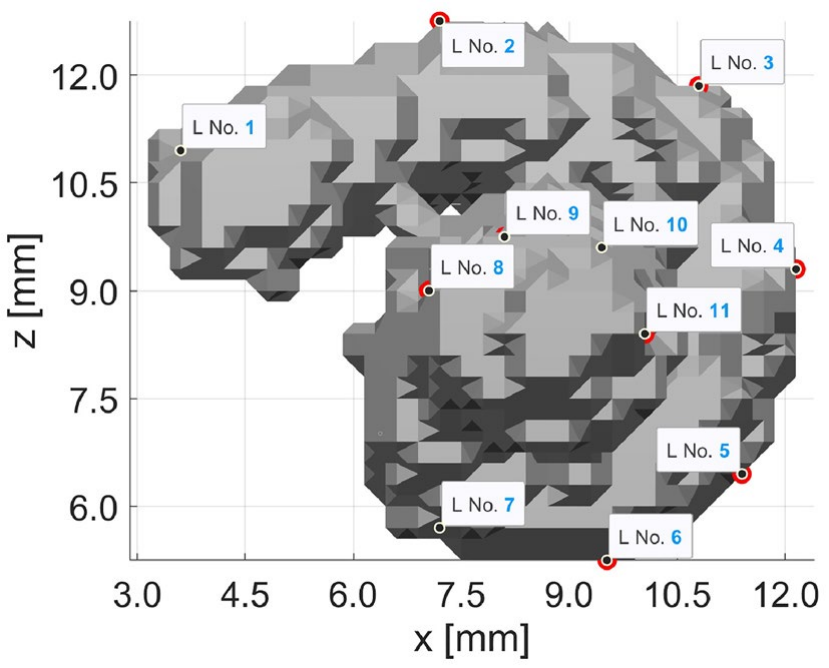
The arrangement of the 11 landmarks of Case 10 are seen, after deleting the 9 landmarks with the largest errors. All these landmarks are located on the second turn of the cochlea.

For this reason, the following investigations in Cases 6 to 12*b* consider whether some of the 20 landmarks present in Case 5 can be omitted. Each step checks which of the landmarks produce the largest error and then deletes them. Initially, a larger step is used, and the three landmarks having the most harmful influence on accuracy in Case 5 are deleted in Case 6. Two landmarks are removed in Cases 7 and 8, while only one landmark is removed in the other cases. With each deleted landmark, the MAE becomes smaller, as seen in Table 1. In Fig. 4 shows the registration results of all these cases, most of which have only changed minimally. Especially worth mentioning is Case 10, which now has 11 landmarks, similar to Case 3. The newly adapted positions of the landmarks can be seen in Fig. 8. Here, it is especially noticeable that landmarks were removed almost exclusively during the second turn. Precisely in this area, setting the landmarks was challenging. In addition, the most substantial deviations in the cochlear segmentation are on the second turn. Determining which voxels still belong to the cochlea and which represent other tissue is tricky in this area. After all, due to the poor resolution, the cochlear boundaries cannot always be clearly identified, which may cause considerable inaccuracies. At the same time, the motivation for registration with the cochlear model is precisely to compensate for such problems within the data. It would be advisable to trust the well-researched cochlear model more than the low-resolution image data. Visually, in the second turn area, the cochlear model and the segmented cochlea do not overlap entirely, either. However, due to the poor resolution of the CT data, it is uncertain whether there is in fact no segmentation error here. Therefore, the results will only be deemed good if the visual results are satisfactory on top of an acceptable error value.

Also, one additional landmark was removed in Case 11 and Cases 12*a* and 12*b*. In Case 11, the landmark designated as No. 8 in Case 10 was removed. This seamlessly follows the other already removed landmarks. Again, no substantial change is visible in the result. Furthermore, in Case 12*a*, the last landmark was removed at the apex of the last turn, which markedly influenced the results. Unsurprisingly, this landmark can lead to a substantial error because it is challenging to detect the boundaries of the cochlea, and a deviation by only a single voxel can have a large effect. However, correctly defining the last landmark is important to map the cochlea’s height correctly. For Case 12*a*, Fig. 4 shows that the transformed model has a slightly lower height than the segmented cochlea. The effect is not that large since there are other landmarks on the last turn. Nevertheless, this is the reason for reinserting the last landmark and removing the landmark from Case 11 (landmark No. 2) instead, which causes the second largest error. This was done in Case 12*b*. Here, the result is almost identical to Case 11 but with an even lower MAE. Thus, an overall MAE reduction from Case 5 to Case 12*b* of $69.88\%$ could be achieved without markedly degrading the visual registration result.

From the previous experiments, some important conclusions can be established: 1. The first four landmarks must be maintained (learned from Case 4). 2. The landmarks on the second turn are very difficult to set and have the most prominent error values. 3. The last landmark is essential for the correct cochlear height.

With this knowledge, one additional case was evaluated. Case 13 considers the three previously mentioned aspects, using only the 5 essential landmarks, i.e. the four on the lowest turn, and the last landmark at the top. All landmarks in between are omitted. As expected, a small MAE value of 0.0414 mm per landmark could be achieved, which is even smaller than in Case 12*b* with the 9 landmarks. However, compared to the effects of the image resolution, the MAE is already so small that these error differences are negligible. Visually, the result in Fig. 5 definitely shows substantial differences from the previous cases, since the cochlear model deviates more clearly from the segmented cochlea. Nevertheless, the second conclusion must also be taken into account, which states that, especially in the area of the second turn, the placement of the landmarks and the segmentation at these points was a major challenge. The landmarks are placed on the lateral wall, the outer spiral of the segmented cochlea. If this outer spiral is already inadequately identified due to insufficient segmentation, this will also affect the result of landmark-based registration. This supports the conclusion that relying only on these 5 landmarks may be sufficient for any further evaluations.

The question of the quality of the previous segmentation remains. Unfortunately, there is no definitive answer. Still, it is known that there is no segmentation that can be widely applied to various datasets of human cochleae, as there are many different CT scanners in use in clinical practice, which differ with respect to technology and parametrization^16^. For this reason, it would be essential to find another solution without prior segmentation. Therefore, the landmarks should be placed directly in the original CT scan, making it possible to reuse a many of the preprocessing steps, including the manual segmentation presented in the previous work^16^. Suppose the model can be directly fitted into the CT. In that case, this can subsequently be used to detect the cochlear characteristics in an automated way, such as cochlear duct length, basal diameter, or volume.

Precisely this approach of placing the landmarks directly into the CT was also tested. For preprocessing, only the rotation of the 3D dataset was performed to have a better view of the cochlear snail, as described in the previous work^16^. Following this, setting the landmarks is done following the same procedure. The landmarks are set on the outer lateral wall. This was performed in two cases. In Case 14, the landmarks were set as in Case 3, and the number of turns was kept. Only the model parameters were recalculated directly using the CT images. The result of the registration is shown in Fig. 6. Here, a single CT slice (from the front and back) was plotted, located at the level of the first four landmarks. It is easy to see that the landmarks in the second and upper turns are farthest apart and cause the most substantial error. However, the total error of 0.1957 mm per landmark is smaller than in Case 3. Overall, however, the cochlear model fits the human cochlea very well. All CT slices were evaluated for fit, and the results were overall highly satisfactory.

Case 15 replicates Case 13 directly in the CT. All 5 landmark pairs are nearly congruent, and the cochlear model seems to fit the human cochlea quite well (see Fig. 7). The MAE is almost identical to the error from Case 13.

Concerning the placement of landmarks directly in the CT data, the process is a complete success, and the results can certainly be used as a basis for further work. Landmark-based registration directly in the CT dataset is much less costly since the preliminary segmentation steps do not have to be taken. Although landmarking can be error-prone, because one voxel difference makes quite a big difference due to the large image resolution, it is less error-prone than full segmentation. It can be learned relatively quickly, even by non-experts. The challenges of segmentation were discussed in Stritzel et al.^16^ The segmentation results are highly dependent on the preprocessing steps. In the study presented here, no statistical test was used, but rather a successive procedure to find an optimal landmark constellation. It cannot be ruled out that there is an even better constellation than the one identified here. Nevertheless, it should be emphasized that the mean error achieved is below the image resolution so an additional error reduction is negligible. Furthermore, the Pietsch model has a weakness in that the cross-section of the cochlea is represented in a perfectly circular shape, which does not correspond to reality. The error-prone landmark setting in combination with the poor image resolution could also result in errors, such as the start of the cochlear snail not being in the center of the round window. Taking into account the limitations, the cochlear model of Pietsch et al.^9^ shall be used to determine the individual characteristics of the human cochlea after registration. From this, in combination with hearing test results, conclusions may be drawn about the subsequent hearing ability, possibly allowing a preoperative prediction of the postoperative hearing success. Based on the large amount of retrospective patient data that is already available, a basis for a more extensive and in-depth study can be laid.

## Methods

The landmark-based registration is a well-described and commonly used method in the literature. In the subject area of the cochlea, for example, this technique was used by Gunz et al.^22^ in 2012 to compare $\mu$CT images of the bony labyrinth of chimpanzees and humans. Nevertheless, to the best of our knowledge, there is no previous work to date concerning the registration of a cochlear model to conventional CT images of humans. Landmarks can be set either automatically or manually. The automatic algorithmic determination of the landmarks has the advantage of reproducible results. In real-world applications, however, an interactive definition of the locations by the user is often necessary^23^. In this approach, a hybrid variant implemented in Matlab is used, which consists of both a manual landmark placement and an automated part. Two image datasets are always required for landmark-based image registration: On one side, there is the human cochlea inside retrospective, preoperative CT scans recorded with a conventional CT scanner. On the other hand, the cochlear model described by Pietsch et al. in the paper *‘Spiral Form of the Human Cochlea Results from Spatial Constraints’*^9^ is utilized.

**Figure 9:**
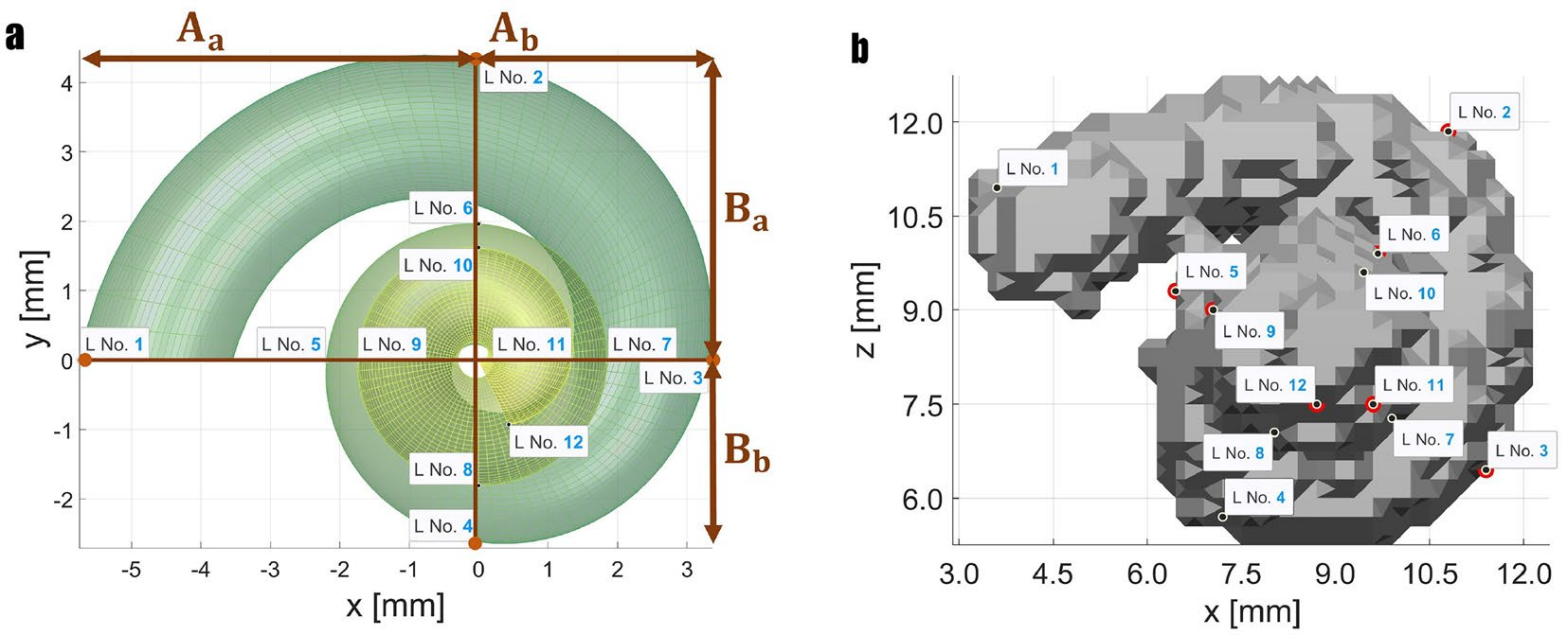
**a** Cochlear model^9^ with mean values. The cochlea can be divided into four quadrants, the length of each quadrant is given by the parameters $A_a$, $A_b$, $B_a$, and $B_b$. Landmark positions are set every $90^{\circ }$ on the cochlear model (**a**) and on the segmented human cochlea, which is slightly rotated here compared to the model (**b**), as in Case 1. There is an unambiguous assignment of the landmark numbers.

In the mentioned publication, a highly precise model of the human cochlea is presented, for which corresponding source code for various languages, including Matlab, is freely available. The cochlea’s center is located in the coordinate origin, i.e. (0, 0, 0), and from this the model is divided into four quadrants. Their sizes are determined by the parameters $A_a$, $A_b$, $B_a$, and $B_b$, which are localized as seen in Fig. 9a. Besides specifying one’s own parameters, creating a mean cochlea is possible. According to Pietsch et al.^9^, the parameters for a mean cochlea are $A_a = 5.97$ mm, $A_b = 3.26$ mm, $B_a = 3.95$ mm, and $B_b = 2.85$ mm. Furthermore, the cochlea’s number of turns is calculated depending on the four previously mentioned parameters.

The conventional CT scans used in this paper have a resolution of 0.3 mm in all three dimensions. During preprocessing, the original human CT data is rotated to reveal the typical snail shape of the cochlea. Subsequently, image enhancement and thresholding are performed, followed by the segmentation of the cochlea as described in Stritzel et al.^16^.In the first experiments, landmarks were placed on the segmented cochlea and later directly into the exclusively rotated CT. The landmarks are placed on the cochlea’s lateral wall, as shown in Fig. 9b. Basically, the number of landmarks that are set is freely selectable. However, the registration result highly depends on the number and position of the landmarks. One landmark is placed every $90^{\circ }$ on the cochlear turn in the first approach. For the mean cochlea, this results in a total number of 12 landmarks. The 12th landmark is positioned at the end of the turn ($<90^\circ$), not necessarily precisely after another $90^{\circ }$. Due to this strict rule of setting landmarks and the well-known cochlear model, automation of this work step is possible for the model.

In human CT scans, positioning the landmarks is more complex and must be done manually. Again, the landmarks are placed on the lateral cochlear wall every $90^{\circ }$. However, due to the resolution, the segmented cochlea does not correspond to an exact spiral shape as in the model. Nevertheless, placing the landmarks as best as possible every $90^{\circ }$ and taking care of a certain regularity is possible. The result of this can be seen in Fig. 9b. The order of the landmarks is essential and must be preserved in any case. A landmark on the model always corresponds to a landmark on the segmented cochlea, marked here with the same number (e.g. L No. 1).

**Figure 10:**
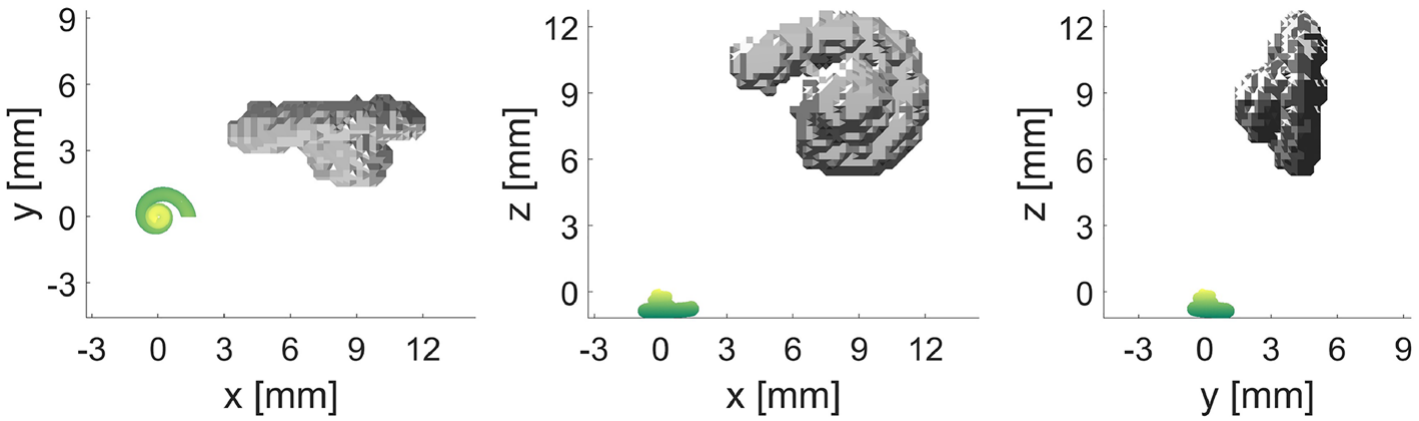
Start positions before registration of cochlear model and segmented cochlea in three different planes. Applies to all cases.

In the next step, the landmarks $\textbf{l}_M$ on the model are to be modified by various transformations so that they fit the corresponding landmarks $\textbf{l}_C$ of the human cochlea. For this purpose, linear transformations, i.e., reflection $f_F$, rotation, and translation $f_{R,t}$ as well as scaling $f_C$ and shearing $f_S$, are executed as composition. The transformation function can thus be described by

1 $$\begin{aligned} \textbf{l}\in \textbf{L}\in \mathbb {R}^{n \times 3},\,\,\,f_T:\,\mathbb {R}^3\rightarrow \mathbb {R}^3,\,\,\, f_T(\textbf{l}) = \bigl ( f_F \circ f_{R,t} \circ f_C \circ f_S \bigr )(\textbf{l}) \end{aligned}$$

with *n* representing the number of landmarks $\textbf{l}$^23^. Since an exact match can usually not be achieved, the total error of the deviations between the corresponding landmarks (sum of squared differences) is minimized. For this purpose, the minimization function

2 $$\begin{aligned} \min _{\mathbf {p^*}} \left\{ \mathscr {F}:= \sum _{i \in \mathbb {N}} \parallel f_T(\textbf{l}_{M,i}, \mathbf {p^*})-\textbf{l}_{C,i}\parallel _2^2 \right\} \,\,\text {with}\,\,\mathbf {p^*} = (\alpha , \beta , \gamma , t_x, t_y, t_z, c_x, c_y, c_z, s_{xy}, s_{xz}, s_{yz}) \end{aligned}$$

is used^23^. There are several different methods in the literature to solve this minimization problem. The Quasi-Newton method was chosen here, although the Levenberg-Marquardt algorithm and the Interior-point method are also possible and provide similarly adequate results^21^.

The algorithm first requires a starting matrix, defined as the unit matrix^21^. At the start time (iteration 0), both the cochlear model and the segmented cochlea have their natural origin shape and origin location, as shown in Fig. 10. The model originates at (0, 0, 0), whereas the origin of the segmented cochlea is clearly located in the positive right quadrant. The original orientations of the two cochleae also differ from each other. However, with each iteration $i+1$ ($i \in \mathbb {N}$), the model increasingly converges to the human cochlea, until minimality is reached. The value $10^{-6}$ is selected as the tolerance threshold for the variation of the sum of squared differences (SSD). This is the standard tolerance for optimization methods in the literature^23^ and the default value in Matlab. After reaching this tolerance threshold, no more notable gains in registration are expected. As a result, the transformation matrix *T* is obtained, transforming the initial cochlear model $M_O$ into the registered model $M_R$ using matrix multiplication $M_R = T \cdot M_O$. If the first termination criterion does not succeed, a maximum number of iterations is performed, which can be freely defined (e.g., 100 iterations). The optimization process is carried out with different numbers of landmarks; see Table 1.

**Figure 11:**
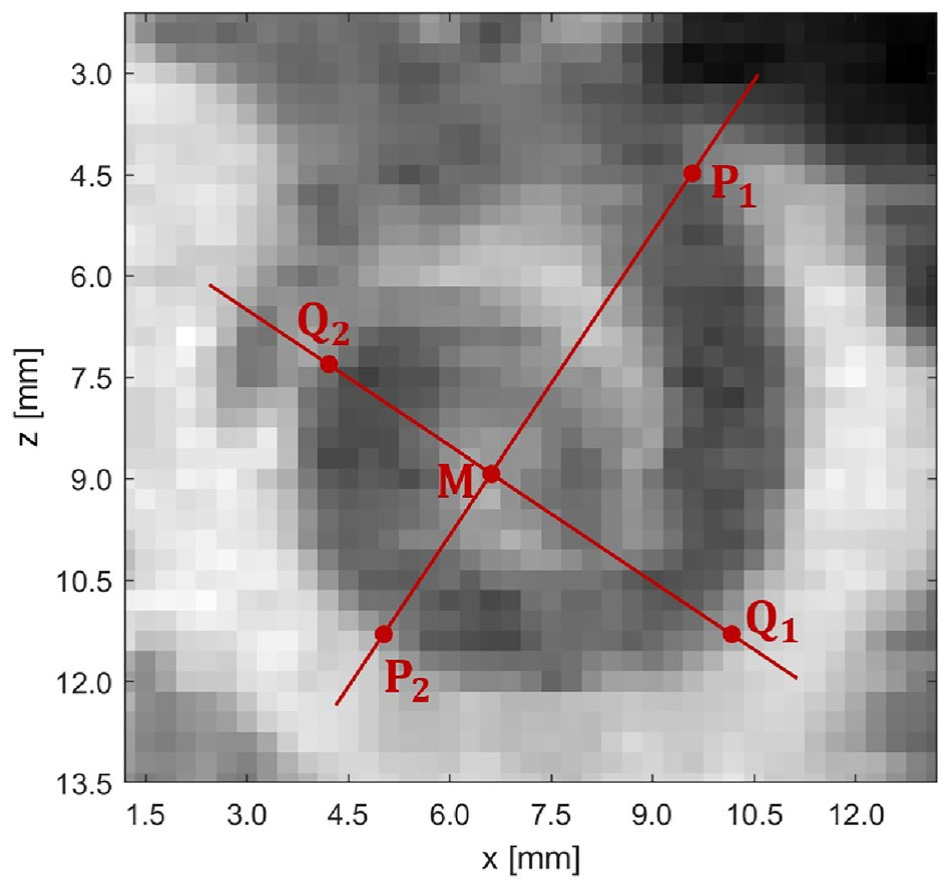
The cochlea can be divided into four quadrants. The length of each quadrant is passed to the cochlear model to create a model that is as close as possible to the real cochlea. This already optimizes the starting point of landmark-based registration.

For obtaining these four parameters, the cochlea is divided into four quadrants whose side lengths correspond to the parameters $A_a,\,A_b,\,B_a,\,B_b$ as shown in Fig. 9 and described in detail in Pietsch et al.^9^. This can be done directly in the CT image, as can be seen in Fig. 11. The human CT data were rotated exactly as described in Stritzel et al.^16^ for better visibility of the spiral shape of the cochlea. In the coronal *x*–*z*-plane, the slice where the cochlea has the largest area in the typical spiral shape is picked out. The outer wall is then the lateral wall on which the landmarks are placed. Here, the problem remains that the resolution of the CT images is insufficient, and the cochlea is difficult to distinguish from the surrounding tissue. The parameters $A_a = \overline{P_1M}$, $A_b = \overline{MP_2}$, $B_a = \overline{Q_1M}$, and $B_b = \overline{MQ_2}$ (in mm) are needed to create the cochlear model, and these are determined by a simple distance calculation. For the first experiments using the segmented cochlea, however, these parameters will be determined directly for the segmented cochlea, as they may differ from those of the original CT image. Only two landmark constellations from the previous experiments are applied directly in the CT. On the one hand, the basic constellation with adjusted parameters and turns, in which the landmarks are set every $90^{\circ }$, and on the other hand, the constellation that previously proved to be the best. Other constellations were previously excluded for various reasons, as they produced errors that were too large, for example due to excessive landmark distances. In the model, the number of turns is calculated as a function of the four input parameters so that it can be adjusted in advance by setting these parameters or detached from the four parameters by setting a fixed value.

In the selected region of interest, landmarks can be placed most effectively by considering the view of all three anatomical planes (axial, coronal, sagittal). This can easily be implemented in Matlab, for example. Otherwise, the landmarks are set according to the same scheme described before, so corresponding landmarks are created again.

### Ethics declarations

The retrospective patient data from clinical routine were processed anonymously, and all patients gave informed consent to participate in scientific evaluations. The Ethics Committee of Hannover Medical School has approved the publication of data collected in clinical routine in an anonymized form (ID 1897–2013). The patient considered here is an adult. We confirm that Regulation (EU) 2016/679 (General Data Protection Regulation) has been followed, and all work was performed in accordance with the Declaration of Helsinki.

## Data Availability

The data supporting this study’s findings are available from the German Hearing Center Hannover, but restrictions apply with respect to the availability of the datasets. They were used under license for the current study. For this reason, the datasets are not publicly available but will be provided by the authors upon reasonable request and with permission of the German Hearing Center Hannover. Please get in touch with the corresponding author for access.
